# Water quality assessment for groundwater around a municipal waste dumpsite

**DOI:** 10.1016/j.dib.2018.01.072

**Published:** 2018-02-01

**Authors:** Olusola T. Kayode, Hilary I. Okagbue, Justina A. Achuka

**Affiliations:** a Department of Physics, Covenant University, Ota, Nigeria; b Department of Mathematics, Covenant University, Ota, Nigeria

**Keywords:** Dumpsite, Environment, Groundwater, Geochemical analysis, Waste, Water, Geostatistics

## Abstract

The dataset for this article contains geostatistical analysis of the level to which groundwater quality around a municipal waste dumpsite located in Oke-Afa, Oshodi/Isolo area of Lagos state, southwestern has been compromised for drinking. Groundwater samples were collected from eight hand-dug wells and two borehole wells around or near the dumpsite. The pH, turbidity, salinity, conductivity, total hydrocarbon, total dissolved solids (TDS), dissolved oxygen, chloride, Sulphate (SO_4_), Nitrate (NO_3_) and Phosphate (PO_4_) were determined for the water samples and compared with World Health Organization (WHO) drinking water standard. Notably, the turbidity, TDS, chloride and conductivity of some of the samples were above the WHO acceptable limits. Also, high quantities of heavy metals such as Aluminum and Barium were also present as shown from the data. The dataset can provide insights into the health implications of the contaminants especially when the mean concentration levels of the contaminants are above the recommended WHO drinking water standard.

## Specification table

**Table:** 

**Subject area**	Earth and Planetary science
**More specific subject area**	Environmental Science, geochemistry, geostatistics
**Type of data**	Table and Figure
**How data was acquired**	pH-conductivity-TDS meter (COMBO HI model 98130), DO-meter (HACH model), ultraviolet (UV)-Visible Spectrophotometer (Camspec model).
**Data format**	Raw, Analysed
**Experimental factors**	The mentioned parameters above, in the abstract section, were analyzed according to the WHO standards for drinking water
**Experimental features**	Determination of physical and chemical parameters that constitute the contaminations of the water near the dumpsites.
**Data source location**	Oke-afa, Oshodi/Isolo area of Lagos State, South-western Nigeria
**Data accessibility**	All the data are in this data article.

## Value of the data

• The data could be used to determine the level of chemical contamination dumpsites, volcanic erupted areas, chemical wastes sites, oil spillage sites and others areas of interest.
• The data could be helpful for concerned authorities and policy makers in water quality management.
• Findings can be extended to other metal or non-metal elements not considered in this article.
• The data could be used in auditing water quality.

## 1 Data

The data contains geostatistical and geochemical analysis of groundwater samples collected from eight (8) hand-dug wells and some borehole wells around or near the dumpsite. The dumpsites are located in Oshodi/Isolo area of Lagos State, South-western Nigeria. The parameters investigated are: pH, dissolved oxygen (DO), chlorine content (CC), total hardness content (THC), salinity, sulphate ($SO_{4}$), Nitrate ($NO_{3}$), Phosphate ($PO_{4}$), conductivity, total dissolved solids (TDS), turbidity, temperature and static water level (SWL). The static water level is not applicable to the two borehole wells. The results of the physio-chemical characteristic of the studied area are presented in Table 1. Results of the heavy metal analysis are presented in Table 2. The detailed descriptive statistics are presented in Table 3. Different measures of central tendency were compared with the WHO recommended limit and this is presented in Table 4.

**Table 1:** The physio-chemical characteristic of groundwater at the dumpsite.

Parameters	W1	W2	W3	W4	W5	W6	W7	W8	W9	W10
pH	6.55	5.15	6.35	6.26	6.59	6.17	6.26	6.25	6.89	6.17
DO mg/l	4.4	4.2	4.3	4.1	4.2	4.0	4.3	4.1	4.0	4.2
CC mg l	92	108	116	44	200	84	40	168	88	344
THC mg/l	200	140	236	240	228	188	120	164	652	428
Salinity mg l	0.18	0.22	0.23	0.09	0.40	0.17	0.08	0.34	0.18	0.69
$SO_{4}$ mg/l	0.07	0.09	1.21	1.27	1.01	0.06	0.08	0.05	2.09	2.12
$NO_{3}$ mg/l	1.20	2.30	2.50	2.60	1.90	2.20	1.76	1.24	3.50	2.90
$PO_{4}$ mg/l	0.09	0.06	1.20	0.10	0.70	0.05	2.10	1.70	3.20	3.00
Conduct mS/cm	952	454	954	1014	1151	994	1007	1120	1643	1123
TDS mg/l	480	211	388	249	573	496	504	561	822	399
Turbidity (NTU)	4.5	2.7	2.9	1.5	3.2	6.9	2.2	2.9	6.9	6.5
Temp (°C)	28.2	27.9	28.3	28.4	28.4	28.3	28.2	29.9	29.2	27.2
SWL m	8	N/A	6	8.6	13	5	6	N/A	2	4

W represents the sample (well and borehole), N.A means Not applicable, W2 and W8 are boreholes.

**Table 2:** Results for the heavy metals analysed on the 10 water samples (Acme Lab Canada).

	Analyte	Dilution	Al	As	Au	B	Ba	Be	Br	Ca	Cd	Ce
	Unit		ppb	ppb	ppb	ppb	ppb	ppb	Ppb	ppm	Ppb	ppb
	MDL	1	1	0.5	0.05	5	0.05	0.05	5	0.05	0.05	0.01
	WHO (ppb)		200	50	–	300	2000	–	25	–	5	–
SOLA 1	Water	1	1	< 0.5	0.14	343	29.05	< 0.05	370	63.05	0.06	< 0.01
SOLA 2	Water	1	25	0.7	< 0.05	90	42.46	0.09	482	36.21	0.28	0.13
SOLA 3	Water	1	13	0.9	< 0.05	177	33.15	< 0.05	700	80.74	0.06	0.06
SOLA 4	Water	1	10	0.5	< 0.05	117	38.28	< 0.05	278	93.94	0.10	0.05
SOLA 5	Water	1	1641	1.4	< 0.05	20	203.4	0.33	371	48.84	0.23	95.07
SOLA 6	Water	1	13	1.0	< 0.05	149	32.92	< 0.05	266	61.60	< 0.05	0.35
SOLA 7	Water	1	89	0.8	< 0.05	172	33.08	0.07	138	39.70	< 0.05	0.22
SOLA 8	Water	1	26	1.1	< 0.05	61	127.5	0.08	890	48.95	0.14	14.93
SOLA 9	Water	1	7	1.4	< 0.05	1438	76.14	< 0.05	269	56.78	< 0.05	0.78
SOLA 10	Water	1	18	4.2	< 0.05	2063	116.8	< 0.05	3547	82.20	< 0.05	0.25
			1–1641	0.5–4.2	0.2	20–2063	29–203.4	0.07–0.33	138–3547	36.21–93.94	0.06–0.24	0.05–95.07
			184.3	1.2	6.45	463	73.28	0.06	731	61.20	0.087	11.18
			486.1	1.075	0.1	664	55.46	0.1	963	18.19	0.096	28.3

Al – Aluminium, As – Arsenic, Au – Gold, B – Boron, Ba – Barium, Br – Bromide, Be – Beryllium, Ca – Calcium, Cd – Cadmium, Ce – Cerium, MDL – MAXIMUM DETECTION LIMIT.

**Table 3:** The descriptive statistics of the parameters of the data.

Parameters	Mean	Standard error	Median	Standard deviation	Variance	Kurtosis	Skewness	Range	Min	Max	Sum
pH	6.26	0.14	6.26	0.45	0.21	4.54	− 1.61	1.74	5.15	6.89	62.64
DO mg/l	4.18	0.04	4.20	0.13	0.02	− 0.75	0.09	0.40	4.00	4.40	41.80
CC mg l	128.40	28.60	100	90.44	8179.38	3.20	1.68	304	40	344	1284
THC mg/l	259.60	51.20	214	161.90	26,209.60	3.67	1.94	532	120	652	2696
Salinity mg l	0.26	0.06	0.20	0.18	0.03	3.19	1.68	0.61	0.08	0.69	2.58
$SO_{4}$ mg/l	0.81	0.27	0.55	0.85	0.72	− 1.31	0.58	2.07	0.05	2.12	8.05
$NO_{3}$ mg/l	2.21	0.23	2.25	0.72	0.52	− 0.24	0.19	2.30	1.20	3.50	22.10
$PO_{4}$ mg/l	1.22	0.39	0.95	1.23	1.51	− 1.19	0.60	3.15	0.05	3.2	12.2
Conduct mS/cm	104.12	91.39	1010.50	288.99	83,513.50	3.50	0.09	1189	454	1643	10,412
TDS mg/l	468.30	55.01	488	173.97	30,264.90	1.05	0.47	611	211	822	4683
Turbidity (NTU)	4.02	0.65	3.05	2.04	4.17	− 1.41	0.58	5.40	1.50	6.90	40.20
Temp (°C)	28.40	0.23	28.3	0.72	0.52	1.80	0.75	2.70	27.2	29.2	284

**Tablele 4:** Comparison of the central tendency estimates with the WHO recommended limits.

Parameters	WHO limit (2008)	Mean	Median	5% Trimmed mean	HuME	TBW	HaME	AW
pH	6.5–8	6.26	6.26	6.29	6.28	6.27	6.30	6.27
DO mg/l	–	4.18	4.20	4.17	4.19	4.18	4.18	4.18
CC mg l	250	128.40	100	121.33	107.10	97.73	102.21	97.95
THC mg/l	500	259.60	214	245.56	209.54	191.66	194.57	191.66
Salinity mg l	–	0.26	0.20	0.24	0.21	0.20	0.20	0.20
$SO_{4}$ mg/l	500	0.81	0.55	0.77	0.65	0.62	0.69	0.63
$NO_{3}$ mg/l	50	2.21	2.25	2.19	2.21	2.19	2.21	2.19
$PO_{4}$ mg/l	0.06	1.22	0.95	1.18	1.04	1.08	1.12	1.08
Conduct mS/cm	500	104.12	1010.50	1040.39	1038.82	1036.73	1038.27	1036.69
TDS mg/l	600	468.30	488	462.94	469.29	452.74	461.95	450.01
Turbidity (NTU)	4.0	4.02	3.05	4	3.58	3.47	3.71	3.48
Temp (°C)	28	28.40	28.3	28.38	28.30	28.29	28.28	28.30

HuME is the Huber's M-Estimator, TBW is the Tukey's bi-weight, HaME is the Hampel's M-Estimator, AW is the Andrew's wave.

## 2 Experimental design, methods and materials

Several data analysis has been carried out on the physio-chemical, geochemical and geostatistical assessment of quality of groundwater [1], [2], [3], [4], [5], [6], [7], [8], [9], [10], [11], [12], [13], [14], [15], [16].

### 2.1 Study qrea and wample collection

**Fig. 1:**
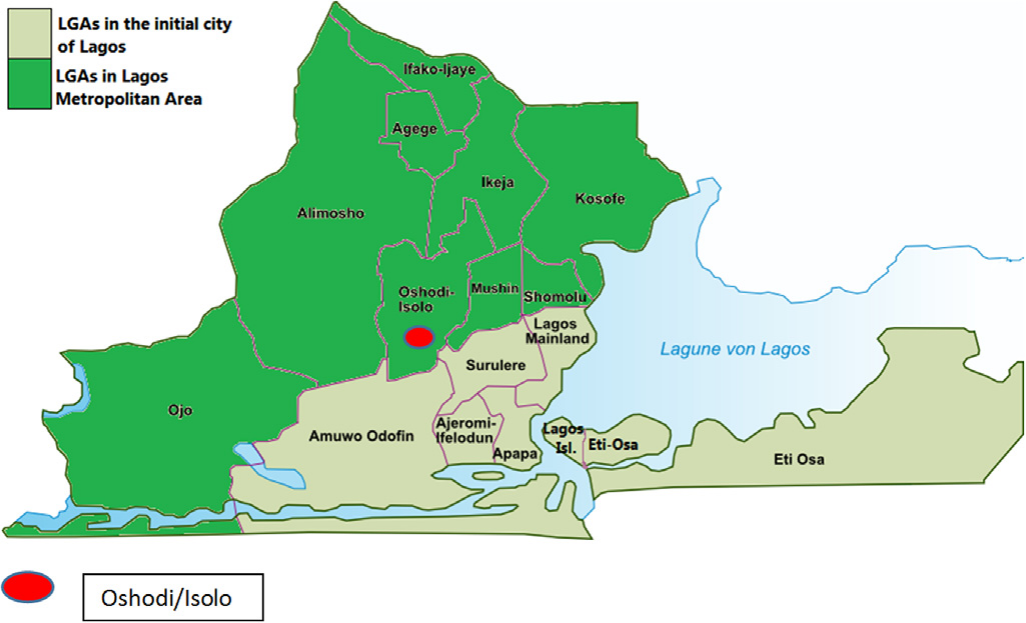
Map of Lagos showing the study area.

**Fig. 2:**
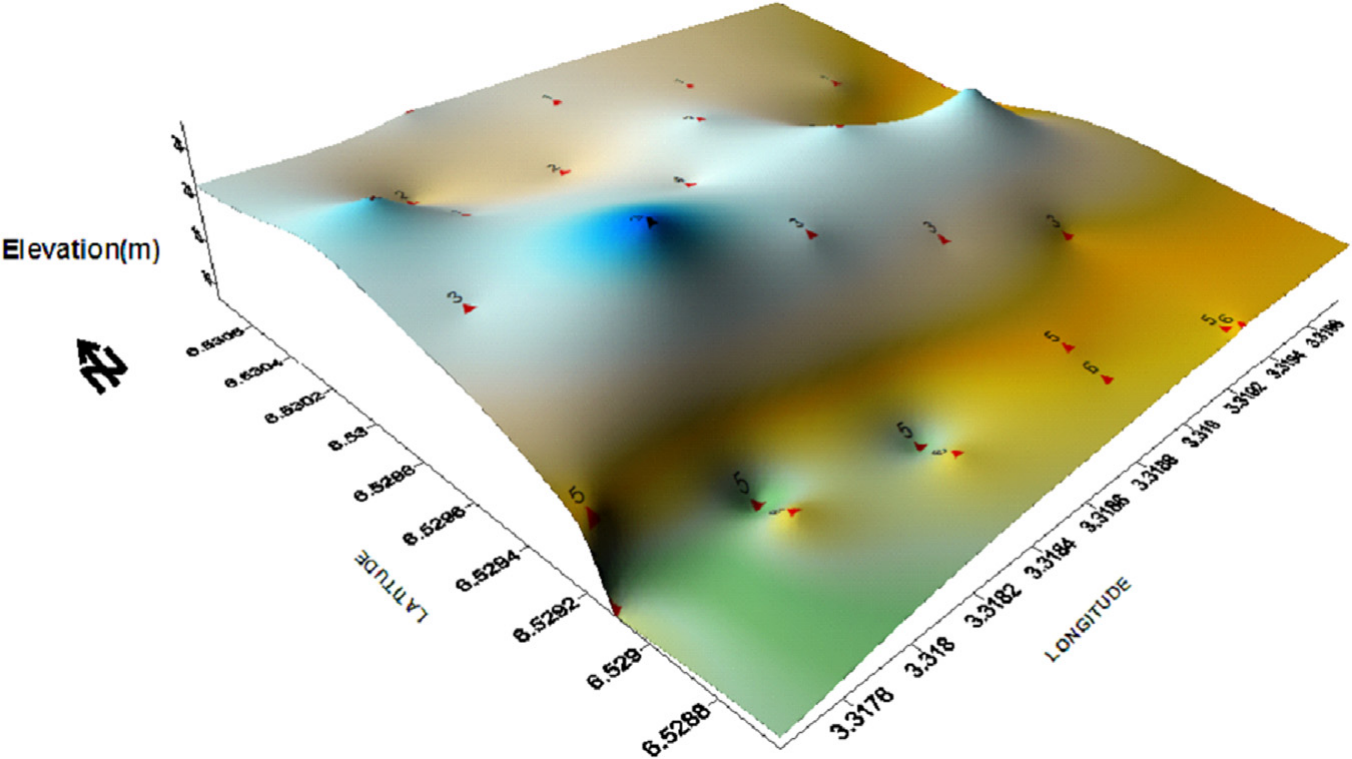
GPS elevation map of the study area.

The data was collected from the areas located around the dumpsite. The dumpsite is an extensive one which has been in existence in Oke-afa, Oshodi/Isolo Area of Lagos State for more than two decades. The detailed GPS coordinates elevation and distance from the dumpsite is presented in Table 5 while the map and GPS elevation map of the studied area can be seen in Figs. 1 and 2 respectively. The boreholes and hand dug wells around this dumpsite had been contaminated by the leachates from the dumpsite.

**Table 5:** GPS Readings and elevation from the 10 hand dug wells and boreholes.

Samples	Latitude	Longitude	Distance from dumpsite (m)	Elevation (m)	**Water table (m)**
W1	N06.52889	E003.31986	10	13.0	8
W2	N06.31471	E003.13073	4	17	N/A
W3	N06.52916	E003.31974	15	18.3	6
W4	N06.52955	E003.31991	30	8.6	8.6
W5	N06.52954	E003.32027	40	12.3	13
W6	N06.52988	E003.32026	35	15.1	5
W7	N06.52998	E003.31994	25	20.1	6
W8	N06.31512	E003.19057	250	18	N/A
W9	N06.31523	E003.19005	55	5	2
W10	N06.3521	E003.19014	300	30	4

Lagos is a sedimentary area located within the western Nigeria coastal zone, a zone of coastal creeks and lagoons developed by barrier beaches associated with sand deposition [17]. The subsurface geology reveals two basic lithologies, clay and sand deposits. These deposits may be inter-bedded in places with sandy clay or clayey sand and occasional with vegetable remains and peat. Basically, the geological setting of the study area reveals that it lies solely within the extensive Dahomey basin, the basin extending almost from Accra to Lagos. The coastal belt varies from about 8 km near the republic of Benin border to 24 km towards the eastern end of the Lagos lagoon [18].

### 2.2 Samples preparation

The samples were collected during the dry season when the demand for water is high due to the hot weather. The residents have both hand dug wells and boreholes but patronize commercial water for drinking purposes. The samples were collected and taken to laboratory for procedural analysis. The pH, conductivity and total dissolved solid (TDS) were measured with pH-conductivity-TDS meter (COMBO HI model 98130). Dissolved oxygen (DO) was measured using DO-meter (HACH model). Anions like sulphate (SO_4_), phosphate (PO_4_), and nitrates (NO_3_) were determined using ultraviolet (UV)-Visible Spectrophotometer (Camspec model). Turbid metric method was used for sulphate determination; Vanado-Molybdo-Phosphoric acid method was used for phosphate determination, while salicylate method was used for nitrate determination. The ${\mathrm{Cl}}^{-}$ concentration was determined by Mohr's method, while hydrocarbonate was determined by titration against 0.01 M of $H_{2}SO_{4}$ using mixed indicator (Bromocresol green-Methyl red solution). The heavy metals in the water samples were analyzed using inductively coupled plasma mass spectrometry (ICPMS) in ACME Laboratory, Canada.

### 2.3 Normality tests

Normality tests are conducted to determine if the observed values are drawn from the normal distribution since the sample size is small. The result is presented in Table 6.

**Table 6:** Normality test of the parameters.

	Kolmogorov–Smirnov			Shapiro–Wilk		
	Statistic	D*f*	Sig.	Statistic	D*f*	Sig.
pH	0.318	10	0.005	0.820	10	0.025
DO mg/l	0.160	10	0.200	0.942	10	0.575
CC mg l	0.255	10	0.065	0.834	10	0.038
THC mg/l	0.348	10	0.001	0.759	10	0.005
Salinity mg l	0.261	10	0.051	0.833	10	0.036
$SO_{4}$ mg/l	0.300	10	0.011	0.805	10	0.017
$NO_{3}$ mg/l	0.112	10	0.200	0.971	10	0.898
$PO_{4}$ mg/l	0.219	10	0.192	0.860	10	0.077
Conduct mS/cm	0.279	10	0.027	0.851	10	0.059
TDS mg/l	0.174	10	0.200	0.946	10	0.627
Turbidity (NTU)	0.256	10	0.062	0.855	10	0.067
Temp (°C)	0.300	10	0.011	0.893	10	0.182

D*f* is the degrees of freedom, Sig is the statistical significance measured as *p*-value.

### 2.4 Correlation coefficient

Correlation among the parameters is conducted to determine the extent of relationship and these are presented in Table 7, Table 8, Table 9.

**Table 7:** Correlation matrix (Pearson).

Variables	pH	DO	CC	THC	Salinity	$SO_{4}$	$NO_{3}$	$PO_{4}$	Conduct	TDS	Turbid	Temp
pH	1	− 0.073	− 0.018	0.519	− 0.023	0.419	0.100	0.394	0.874	0.764	0.309	0.372
DO		1	0.038	− 0.422	0.026	− 0.256	− 0.489	− 0.163	− 0.427	− 0.319	− 0.407	− 0.421
CC			1	0.277	0.999	0.449	0.128	0.419	0.119	0.013	0.351	− 0.325
THC				1	0.281	0.853	0.787	0.690	0.772	0.569	0.650	0.074
Salinity					1	0.451	0.134	0.422	0.121	0.014	0.353	− 0.320
$SO_{4}$						1	0.827	0.614	0.607	0.232	0.370	− 0.181
$NO_{3}$							1	0.464	0.396	0.112	0.428	− 0.221
$PO_{4}$								1	0.674	0.571	0.392	0.115
Conduct									1	0.854	0.486	0.444
TDS										1	0.519	0.558
Turbid											1	− 0.121
Temp												1

**Table 8:** Correlation matrix (Spearman).

Variables	pH	DO	CC	THC	Salinity	$SO_{4}$	$NO_{3}$	$PO_{4}$	Conduct	TDS	Turbid	Temp
pH	1	0.134	− 0.067	0.445	− 0.056	0.238	0.000	0.360	0.427	0.573	0.187	0.495
DO		1	0.136	− 0.285	0.084	− 0.062	− 0.446	− 0.031	− 0.495	− 0.303	− 0.343	− 0.563
CC			1	0.248	0.997	0.188	0.042	0.200	0.248	0.042	0.274	− 0.073
THC				1	0.280	0.806	0.745	0.418	0.564	0.079	0.421	0.208
Salinity					1	0.225	0.097	0.243	0.298	0.073	0.291	− 0.040
$SO_{4}$						1	0.867	0.503	0.442	− 0.164	0.073	− 0.153
$NO_{3}$							1	0.370	0.382	− 0.188	0.189	− 0.018
$PO_{4}$								1	0.697	0.479	0.116	0.183
Conduct									1	0.697	0.323	0.526
TDS										1	0.476	0.581
Turbid											1	0.037
Temp												1

**Table 9:** Correlation matrix (Kendall).

Variables	pH	DO	CC	THC	Salinity	$SO_{4}$	$NO_{3}$	$PO_{4}$	Conduct	TDS	Turbid	Temp
pH	1	0.122	− 0.068	0.296	− 0.046	0.159	− 0.023	0.205	0.250	0.477	0.163	0.376
DO		1	0.072	− 0.263	0.048	− 0.119	− 0.358	− 0.024	− 0.358	− 0.214	− 0.244	− 0.395
CC			1	0.200	0.989	0.156	0.067	0.156	0.156	0.022	0.205	− 0.046
THC				1	0.225	0.689	0.600	0.333	0.422	0.022	0.341	0.184
Salinity					1	0.180	0.090	0.180	0.180	0.045	0.230	− 0.023
$SO_{4}$						1	0.733	0.378	0.289	− 0.111	0.023	− 0.092
$NO_{3}$							1	0.289	0.289	− 0.111	0.114	0.000
$PO_{4}$								1	0.556	0.333	0.068	0.138
Conduct									1	0.600	0.250	0.460
TDS										1	0.386	0.414
Turbid											1	0.000
Temp												1

In order for better understanding of the correlations, the distances between the correlations are computed using the following;

$$D_{1}=\left|Pearson-Spearman\right|$$

${\;D}_{2}=\left|Kendall-Pearson\right|$

${\;D}_{3}=\left|Spearman-Kendall\right|$

The application of the transformations and their percentages using Table 7, Table 8, Table 9 are presented in Table 10.

**Table 10:** Absolute difference between the correlations coefficients and their percentages.

Variables	$D_{1}$	$D_{2}$	$D_{3}$	$\%D_{1}$	$\%D_{2}$	$\%D_{3}$
1	0.207	0.195	0.012	20.7	19.5	1.2
2	0.049	0.050	0.001	4.9	5.0	0.1
3	0.074	0.223	0.149	7.4	22.3	14.9
4	0.033	0.023	0.010	3.3	2.3	1.0
5	0.181	0.260	0.079	18.1	26.0	7.9
6	0.100	0.123	0.023	10.0	12.3	2.3
7	0.034	0.189	0.155	3.4	18.9	15.5
8	0.447	0.624	0.177	44.7	62.4	17.7
9	0.191	0.287	0.096	19.1	28.7	9.6
10	0.122	0.146	0.024	12.2	14.6	2.4
11	0.123	0.004	0.119	12.3	0.4	11.9
12	0.098	0.034	0.064	9.8	3.4	6.4
13	0.137	0.159	0.022	13.7	15.9	2.2
14	0.058	0.022	0.036	5.8	2.2	3.6
15	0.194	0.137	0.057	19.4	13.7	5.7
16	0.043	0.131	0.088	4.3	13.1	8.8
17	0.132	0.139	0.007	13.2	13.9	0.7
18	0.068	0.069	0.137	6.8	6.9	13.7
19	0.016	0.105	0.089	1.6	10.5	8.9
20	0.064	0.163	0.099	6.4	16.3	9.9
21	0.142	0.026	0.168	14.2	2.6	16.8
22	0.029	0.077	0.048	2.9	7.7	4.8
23	0.002	0.010	0.008	0.2	1.0	0.8
24	0.261	0.293	0.032	26.1	29.3	3.2
25	0.086	0.061	0.025	8.6	6.1	2.5
26	0.219	0.263	0.044	21.9	26.3	4.4
27	0.129	0.037	0.092	12.9	3.7	9.2
28	0.029	0.009	0.020	2.9	0.9	2.0
29	0.077	0.146	0.069	7.7	14.6	6.9
30	0.252	0.279	0.027	25.2	27.9	2.7
31	0.001	0.056	0.055	0.1	5.6	5.5
32	0.047	0.164	0.117	4.7	16.4	11.7
33	0.042	0.187	0.145	4.2	18.7	14.5
34	0.272	0.357	0.085	27.2	35.7	8.5
35	0.208	0.350	0.142	20.8	35.0	14.2
36	0.490	0.547	0.057	49.0	54.7	5.7
37	0.229	0.309	0.080	22.9	30.9	8.0
38	0.134	0.110	0.024	13.4	11.0	2.4
39	0.226	0.271	0.045	22.6	27.1	4.5
40	0.037	0.044	0.007	3.7	4.4	0.7
41	0.179	0.242	0.063	17.9	24.2	6.3
42	0.177	0.059	0.118	17.7	5.9	11.8
43	0.059	0.031	0.028	5.9	3.1	2.8
44	0.062	0.123	0.061	6.2	12.3	6.1
45	0.280	0.297	0.017	28.0	29.7	1.7
46	0.040	0.094	0.134	4.0	9.4	13.4
47	0.111	0.236	0.125	11.1	23.6	12.5
48	0.165	0.318	0.153	16.5	31.8	15.3
49	0.396	0.343	0.053	39.6	34.3	5.3
50	0.297	0.347	0.050	29.7	34.7	5.0
51	0.028	0.089	0.061	2.8	8.9	6.1
52	0.094	0.175	0.081	9.4	17.5	8.1
53	0.014	0.107	0.093	1.4	10.7	9.3
54	0.300	0.223	0.077	30.0	22.3	7.7
55	0.239	0.314	0.075	23.9	31.4	7.5
56	0.203	0.221	0.018	20.3	22.1	1.8
57	0.023	0.118	0.141	2.3	11.8	14.1
58	0.092	0.238	0.146	9.2	23.8	14.6
59	0.276	0.324	0.048	27.6	32.4	4.8
60	0.068	0.023	0.045	6.8	2.3	4.5
61	0.157	0.254	0.097	15.7	25.4	9.7
62	0.163	0.236	0.073	16.3	23.6	7.3
63	0.082	0.016	0.066	8.2	1.6	6.6
64	0.043	0.133	0.090	4.3	13.3	9.0
65	0.023	0.144	0.167	2.3	14.4	16.7
66	0.158	0.121	0.037	15.8	12.1	3.7

The variables are the correlations between the parameters.

### 2.5 Analysis of variance

The result showed that there are significant differences in the means of the parameters that constitute contamination of the 10 samples collected from the study area. This is presented in Table 11.

**Table 11:** Analysis of variance (ANOVA) for the samples.

Source of variation	D.F	S.S	M.S	*F*-value	*P*-value
Sample	11	10,729,618	975,419.9	78.99464	< 0.0000005
Error	108	1,333,576	12,347.92		
Total	119	12,063,194			
